# Dietary Intake of Tomato and Lycopene and Risk of All-Cause and Cause-Specific Mortality: Results From a Prospective Study

**DOI:** 10.3389/fnut.2021.684859

**Published:** 2021-07-05

**Authors:** Xin Xu, Shiqi Li, Yi Zhu

**Affiliations:** Department of Urology, The First Affiliated Hospital, School of Medicine, Zhejiang University, Hangzhou, China

**Keywords:** tomato, lycopene, mortality, cohort, PLCO

## Abstract

Evidence on the relationship between consumption of tomato or lycopene and mortality is limited. We investigated the associations of raw tomato, tomato catsup or lycopene intake with all-cause and cause-specific mortality using data from the Prostate, Lung, Colorectal, and Ovarian (PLCO) screening trial. A multivariate Cox proportional hazards model was used to estimate hazard ratios (HRs) and 95% confidence intervals (CIs). During a total of 1,672,715 follow-up years, 24,141 all-cause deaths, 7,534 cardiovascular disease (CVD) deaths and 7,161 cancer deaths occurred. Total mortality was statistically significantly inversely associated with intake of raw tomato (Q5 vs. Q1; HR, 0.95 [95% CI, 0.91–0.99]), tomato catsup (Q5 vs. Q1; HR, 0.93 [95% CI, 0.89–0.97]), and moderate lycopene (Q4 vs. Q1; HR, 0.88 [95% CI, 0.85–0.93]). CVD mortality was significantly inversely related with intake of moderate raw tomato (Q4 vs. Q1; HR, 0.90 [95% CI, 0.83–0.97]), tomato catsup (Q5 vs. Q1; HR, 0.92 [95% CI, 0.85–0.99]), and moderate lycopene (Q4 vs. Q1; HR, 0.90 [95% CI, 0.83–0.98]). Dietary intake of raw tomato (Q5 vs. Q1; HR, 1.04 [95% CI, 0.96–1.14]) and tomato catsup (Q5 vs. Q1; HR, 1.00 [95% CI, 0.93–1.08]) were not related with cancer mortality. Moderate dietary intake of lycopene was significantly associated with a lower cancer mortality (Q4 vs. Q1; HR, 0.89 [95% CI, 0.82–0.96]). There was a non-linear J-shaped association between consumption of raw tomato, tomato catsup or lycopene and total mortality (*P* for non-linearity <0.001). In conclusion, in this large nationally representative sample of US adult population, tomato products, and lycopene intake were associated with lower risks of total and CVD mortality. Moderate consumption of lycopene was also related with a reduced cancer mortality. Further clinical studies and dietary intervention studies are warranted to confirm our premilitary findings.

## Introduction

Tomato and tomato products have been widely recognized as healthy foods because of their high content of lycopene. Human trials have shown the potential of dietary lycopene or lycopene supplement in reducing levels of proinflammatory mediators and oxidative stress (1), regulating cardiovascular variables (2) and blood pressure (3), and improving the lipid profile (4). Epidemiological studies have shown inverse associations between consumption of tomato or lycopene and the risk of developing several major chronic diseases, including incidence of cardiovascular disease (CVD) (5, 6), metabolic syndrome (7, 8), dementias (9), and some types of cancer (10–13). There is an increasing interest in the association between tomato or lycopene intake and mortality. To the best of our knowledge, currently only one cohort study has been published on this topic, which reported an inverse relationship between tomato intake and both total and CVD mortality (14). However, this study failed to distinguish raw tomatoes and processed tomatoes, which may have differential effects on health outcomes. In addition, this study did not further explore the potential dose-response relationship between tomato or lycopene consumption and mortality.

To provide evidence to bridge this knowledge gap, we investigated the associations of raw tomato, tomato catsup or lycopene intake with all-cause and cause-specific mortality using data from the Prostate, Lung, Colorectal, and Ovarian (PLCO) screening trial.

## Methods

### Subjects and Study Design

The design and methods of the PLCO screening trial have been previously described (15). Briefly, the PLCO study is a randomized, controlled trial to assess whether certain screening tests reduce death from prostate, lung, colorectal, and ovarian cancer. PLCO consisted of ~155,000 participants aged 55–74 years and enrolled between November 1993 and July 2001. The participants were from 10 clinical screening centers throughout the United States. PLCO study was approved by the institutional review boards of the National Cancer Institute and each of the participating centers. Informed consent was obtained from each eligible participant in the study. The ClinicalTrials.gov numbers for PLCO are NCT00002540, NCT01696968, NCT01696981, and NCT01696994.

### Data Collection and Dietary Assessment

All participants were asked to complete a baseline questionnaire (BQ) containing baseline information such as demographics and medical history. The Dietary History Questionnaire (DHQ) was administered to participants to collect dietary data. DHQ included the prespecified portion size and consumption frequency of 124 food items and supplement use over the previous year (16). The USDA 1994–1996 Continuing Survey of Food Intakes by Individuals (17) were used to calibrate DHQ data and calculate the daily intake of tomato products and lycopene.

### Participant Selection

Participants were omitted from this study if they did not complete a BQ (*n* = 4,918); had reported a previous cancer at baseline (*n* = 10,199); did not have follow-up time (*n* = 12); failed to complete DHQ or the DHQ was not valid (*n* = 37,936). Finally, our study included a total of 101,832 individuals.

### Outcome Assessment

Study participants were followed from the date of DHQ completion to the time of death or through 2015. Deaths were identified by the administration of the Annual Study Update (ASU) questionnaires, reports from relatives, friends, or physicians, and National Death Index (NDI) Plus searches. The cause of deaths was classified according to the International Classification of Diseases, 9th Revision (ICD-9). The primary outcomes of interest were all-cause mortality (death from any cause) and mortality from CVD or cancer.

### Statistical Analysis

Tomato or lycopene consumption was categorized into five equal groups. A multivariate Cox proportional hazards model was used to estimate hazard ratios (HRs) and 95% confidence intervals (CIs). Two models were established to adjust for variables. Model 1 was adjusted for age (continuous) and sex (male vs. female). Model 2 was further adjusted for race (non-Hispanic White vs. Other), body mass index (BMI, continuous), education (≤ high school vs. ≥some college), smoking status (never vs. former ≤ 15 years since quit vs. former >15 years since quit vs. former year since quit unknown vs. current smoker ≤ 1 pack per day vs. current smoker >1 pack per day vs. current smoker intensity unknown), marital status (married vs. not married), randomization arm (screening group vs. control group), aspirin use (yes vs. no), history of hypertension (yes vs. no), history of diabetes (yes vs. no), history of stroke (yes vs. no), history of heart attack (yes vs. no), vegetables intake (continuous), fruit intake (continuous), alcohol drinking status (never vs. former vs. current), and total energy intake (continuous).

Subgroup analyses were performed based on sex, smoking status, and BMI. Sensitivity analyses were conducted by excluding events that occurred within 2 years or within 5 years of follow-up. Interaction assessments were tested using likelihood-ratio tests compared models with and without the interaction term. The proportional hazards (PH) assumption was checked using the Schoenfeld residual test (18). Restricted cubic spline models (19) with three fitted knots (i.e., 10th, 50th, and 90th percentiles) were used to investigate the dose-response relationship between tomato or lycopene intake (as a continuous variable) and each outcome after full adjustment. All statistical analyses were performed using the software STATA version 15 (Stata Corp, College Station, TX, USA) with two-sided *P*-values.

## Results

### Study Characteristic

During a total of 1,672,715 follow-up years, 24,141 all-cause deaths, 7,534 CVD deaths and 7,161 cancer deaths occurred. The median (IQR) follow-up duration was 17.0 (15.0–19.0) years. The average age of participants at baseline was 62.4 (*SD* 5.3) years. The median intakes of raw tomato, tomato catsup, and lycopene were 12.91 g/day, 0.58 g/day, and 4.76 mg/day, respectively. In comparison with participants in the lowest category of raw tomato, tomato catsup or lycopene, participants in the highest category were fatter, more likely to be non-Hispanic White and former smokers, and have a higher total energy intake (Table 1 and Supplementary Tables 1, 2).

**Table 1:** Main characteristic of participants included in this study by raw tomato intake.

**Variables**	**Q1 (*n* = 20,508)**	**Q2 (*n* = 20,276)**	**Q3 (*n* = 20,619)**	**Q4 (*n* = 20,928)**	**Q5 (*n* = 19,506)**	***p*-value**
Age (y), mean (*SD*)	62.2 (5.4)	62.4 (5.3)	62.5 (5.3)	62.6 (5.2)	62.4 (5.2)	<0.001
**Sex (*****n*****, %)**						
Male	11,349 (55.3%)	10,239 (50.5%)	9,433 (45.7%)	8,694 (41.5%)	9,818 (50.3%)	<0.001
Female	9,158 (44.7%)	10,037 (49.5%)	11,186 (54.3%)	12,232 (58.5%)	9,686 (49.7%)	
**Smoking status (*****n*****, %)**						
Never	9,380 (45.7%)	9,704 (47.9%)	10,146 (49.2%)	10,289 (49.2%)	9,077 (46.5%)	<0.001
Current	2,358 (11.5%)	1,994 (9.8%)	1,694 (8.2%)	1,710 (8.2%)	1,656 (8.5%)	
Former	8,766 (42.8%)	8,575 (42.3%)	8,770 (42.6%)	8,926 (42.7%)	8,767 (45.0%)	
**Education (*****n*****, %)**						
≤ High school	9,332 (45.5%)	8,767 (43.2%)	8,299 (40.2%)	8,657 (41.4%)	7,912 (40.6%)	<0.001
≥Some college	11,130 (54.3%)	11,460 (56.5%)	12,277 (59.5%)	12,234 (58.5%)	11,560 (59.3%)	
**BMI (*****n*****, %)**						
<25.0 kg/m^2^	6,925 (33.8%)	6,895 (34.0%)	7,154 (34.7%)	7,257 (34.7%)	6,248 (32.0%)	<0.001
≥25.0 kg/m^2^	13,281 (64.8%)	13,104 (64.6%)	13,223 (64.1%)	13,406 (64.1%)	12,990 (66.6%)	
**Race (*****n*****, %)**						
White, non-Hispanic	17,582 (85.7%)	18,341 (90.5%)	19,143 (92.8%)	19,475 (93.1%)	18,056 (92.6%)	<0.001
Other	2,915 (14.2%)	1,928 (9.5%)	1,472 (7.1%)	1,444 (6.9%)	1,439 (7.4%)	
**Alcohol drinking status (*****n*****, %)**						
Never	1,991 (9.7%)	1,997 (9.8%)	2,011 (9.8%)	2,143 (10.2%)	1,982 (10.2%)	<0.001
Former	3,499 (17.1%)	3,010 (14.8%)	2,701 (13.1%)	2,875 (13.7%)	2,684 (13.8%)	
Current	14,359 (70.0%)	14,703 (72.5%)	15,391 (74.6%)	15,305 (73.1%)	14,302 (73.3%)	
Total energy intake (kcal/d), mean (SD)	1554.1 (711.8)	1635.6 (690.4)	1714.1 (683.7)	1784.6 (707.5)	2015.8 (803.6)	<0.001

*Y, year; SD, standard deviation; BMI, body mass index*.

### Tomato or Lycopene Intake and All-Cause Mortality

Based on the most fully adjusted model 2, raw tomato intake was statistically significantly inversely associated with total mortality (Q5 vs. Q1; HR, 0.95 [95% CI, 0.91–0.99]) (Table 2). Higher consumption of tomato catsup was significantly associated with a lower total mortality (Q5 vs. Q1; HR, 0.93 [95% CI, 0.89–0.97]). Moderate dietary intake of lycopene was inversely linked with a reduced total mortality (Q4 vs. Q1; HR, 0.88 [95% CI, 0.85–0.93]).

**Table 2:** Associations between intake of raw tomato, tomato catsup or lycopene, and total mortality.

**Variables**	**Median**	**Cohort (*n*)**	**Cases (*n*)**	**HR (95% CI)xy^#^, *p*-value**	HR (95% CI)^*^, *p*-value
**Raw tomato (g/day)**					
Q1 (≤ 3.63)	1.63	20,508	5,288	Reference group	Reference group
Q2 (≥3.65– ≤ 9.53)	6.33	20,276	4,872	0.91 (0.88–0.95), *p* < 0.001	0.96 (0.92–0.99), *p* = 0.027
Q3 (≥9.55– ≤ 17.56)	12.91	20,619	4,669	0.86 (0.83–0.90), *p* < 0.001	0.93 (0.89–0.96), *p* < 0.001
Q4 (≥17.67– ≤ 32.44)	23.79	20,928	4,658	0.85 (0.82–0.89), *p* < 0.001	0.91 (0.87–0.95), *p* < 0.001
Q5 (≥32.64)	50.24	19,506	4,654	0.91 (0.87–0.94), *p* < 0.001	0.95 (0.91–0.99), *p* = 0.026
				*p* for trend = 0.001	*p* for trend = 0.141
**Tomato catsup (g/day)**					
Q1 (≤ 0.11)	0	21,636	5,501	Reference group	Reference group
Q2 (≥0.13– ≤ 0.44)	0.17	19,575	4,306	0.92 (0.89–0.96), *p* < 0.001	0.94 (0.90–0.98), *p* = 0.004
Q3 (≥0.48– ≤ 1.15)	0.58	21,243	4,540	0.88 (0.85–0.92), *p* < 0.001	0.91 (0.87–0.94), *p* < 0.001
Q4 (≥1.20– ≤ 2.53)	1.99	19,486	5,057	0.91 (0.88–0.95), *p* < 0.001	0.93 (0.90–0.97), *p* = 0.001
Q5 (≥2.95)	5.06	19,897	4,737	0.95 (0.91–0.99), *p* = 0.013	0.93 (0.89–0.97), *p* = 0.001
				*p* for trend = 0.923	*p* for trend = 0.080
**Lycopene (mg/day)**					
Q1 (<2.79)	2.07	20,368	5,417	Reference group	Reference group
Q2 (≥2.79– <4.06)	3.42	20,367	4,722	0.89 (0.86–0.93), *p* < 0.001	0.92 (0.89–0.96), *p* < 0.001
Q3 (≥4.06– <5.61)	4.76	20,368	4,509	0.87 (0.84–0.91), *p* < 0.001	0.91 (0.87–0.95), *p* < 0.001
Q4 (≥5.61– <8.44)	6.74	20,367	4,430	0.87 (0.83–0.90), *p* < 0.001	0.88 (0.85–0.93), *p* < 0.001
Q5 (≥8.44)	12.06	20,367	5,063	1.00 (0.96–1.04), *p* = 0.947	0.99 (0.94–1.04), *p* = 0.627
				*p* for trend = 0.012	*p* for trend = 0.203

# *Adjusted for age (continuous) and sex (male vs. female)*.

* *Further adjusted for race (non-Hispanic White vs. Other), body mass index (BMI, continuous), education (≤ high school vs. ≥some college), smoking status (never vs. former ≤ 15 years since quit vs. former >15 years since quit vs. former year since quit unknown vs. current smoker ≤ 1 pack per day vs. current smoker >1 pack per day vs. current smoker intensity unknown), marital status (married vs. not married), randomization arm (screening group vs. control group), aspirin use (yes vs. no), history of hypertension (yes vs. no), history of diabetes (yes vs. no), history of stroke (yes vs. no), history of heart attack (yes vs. no), vegetables intake (continuous), fruit intake (continuous), alcohol drinking status (never vs. former vs. current), and total energy intake (continuous)*.

### Tomato or Lycopene Intake and Cause-Specific Mortality

Based on the most fully adjusted model 2, moderate dietary intake of raw tomato was statistically significantly associated with a lower CVD mortality (Q4 vs. Q1; HR, 0.90 [95% CI, 0.83–0.97]) (Table 3). Increased consumption of tomato catsup was significantly associated with a reduced CVD mortality (Q5 vs. Q1; HR, 0.92 [95% CI, 0.85–0.99]). Moderate dietary intake of lycopene was significantly related with a lower CVD mortality (Q4 vs. Q1; HR, 0.90 [95% CI, 0.83–0.98]).

**Table 3:** Associations between intake of raw tomato, tomato catsup or lycopene, and CVD mortality.

**Variables**	**Median**	**Cohort (*n*)**	**Cases (*n*)**	**HR (95% CI)^#^, *p*-value**	HR (95% CI)^*^, *p*-value
**Raw tomato (g/day)**					
Q1 (≤ 3.63)	1.63	20,508	1,645	Reference group	Reference group
Q2 (≥3.65– ≤ 9.53)	6.33	20,276	1,526	0.92 (0.86–0.99), *p* = 0.018	0.96 (0.90–1.04), *p* = 0.327
Q3 (≥9.55– ≤ 17.56)	12.91	20,619	1,491	0.89 (0.83–0.95), *p* = 0.001	0.96 (0.90–1.04), *p* = 0.323
Q4 (≥17.67– ≤ 32.44)	23.79	20,928	1,413	0.84 (0.78–0.90), *p* < 0.001	0.90 (0.83–0.97), *p* = 0.006
Q5 (≥32.64)	50.24	19,506	1,459	0.92 (0.86–0.98), *p* = 0.017	0.95 (0.88–1.03), *p* = 0.248
				*p* for trend = 0.063	*p* for trend = 0.268
**Tomato catsup (g/day)**					
Q1 (≤ 0.11)	0	21,636	1,705	Reference group	Reference group
Q2 (≥0.13– ≤ 0.44)	0.17	19,575	1,328	0.93 (0.86–1.00), *p* = 0.045	0.96 (0.89–1.03), *p* = 0.236
Q3 (≥0.48– ≤ 1.15)	0.58	21,243	1,400	0.89 (0.83–0.95), *p* = 0.001	0.91 (0.85–0.98), *p* = 0.009
Q4 (≥1.20– ≤ 2.53)	1.99	19,486	1,634	0.93 (0.87–1.00), *p* = 0.043	0.96 (0.89–1.03), *p* = 0.285
Q5 (≥2.95)	5.06	19,897	1,467	0.95 (0.88–1.02), *p* = 0.140	0.92 (0.85–0.99), *p* = 0.029
				*p* for trend = 0.903	*p* for trend = 0.167
**Lycopene (mg/day)**					
Q1 (<2.79)	2.07	20,368	1,688	Reference group	Reference group
Q2 (≥2.79– <4.06)	3.42	20,367	1,494	0.91 (0.85–0.98), *p* = 0.011	0.95 (0.89–1.03), *p* = 0.200
Q3 (≥4.06– <5.61)	4.76	20,368	1,378	0.87 (0.81–0.94), *p* < 0.001	0.91 (0.85–0.98), *p* = 0.017
Q4 (≥5.61– <8.44)	6.74	20,367	1,373	0.88 (0.82–0.94), *p* < 0.001	0.90 (0.83–0.98), *p* = 0.012
Q5 (≥8.44)	12.06	20,367	1,601	1.03 (0.96–1.11), *p* = 0.375	1.01 (0.93–1.11), *p* = 0.742
				*p* for trend = 0.033	*p* for trend = 0.265

# *Adjusted for age (continuous) and sex (male vs. female)*.

* *Further adjusted for race (non-Hispanic White vs. Other), body mass index (BMI, continuous), education (≤ high school vs. ≥some college), smoking status (never vs. former ≤ 15 years since quit vs. former >15 years since quit vs. former year since quit unknown vs. current smoker ≤ 1 pack per day vs. current smoker >1 pack per day vs. current smoker intensity unknown), marital status (married vs. not married), randomization arm (screening group vs. control group), aspirin use (yes vs. no), history of hypertension (yes vs. no), history of diabetes (yes vs. no), history of stroke (yes vs. no), history of heart attack (yes vs. no), vegetables intake (continuous), fruit intake (continuous), alcohol drinking status (never vs. former vs. current), and total energy intake (continuous)*.

Based on the most fully adjusted model 2, dietary intake of raw tomato was not associated with cancer mortality (Q5 vs. Q1; HR, 1.04 [95% CI, 0.96–1.14]) (Table 4). Higher consumption of tomato catsup was also not related with cancer mortality (Q5 vs. Q1; HR, 1.00 [95% CI, 0.93–1.08]). Moderate dietary intake of lycopene was significantly associated with a lower cancer mortality (Q4 vs. Q1; HR, 0.89 [95% CI, 0.82–0.96]).

**Table 4:** Associations between intake of raw tomato, tomato catsup or lycopene, and cancer mortality.

**Variables**	**Median**	**Cohort (*n*)**	**Cases (*n*)**	**HR (95% CI)^#^, *p*-value**	HR (95% CI)^*^, *p*-value
**Raw tomato (g/day)**					
Q1 (≤ 3.63)	1.63	20,508	1,479	Reference group	Reference group
Q2 (≥3.65– ≤ 9.53)	6.33	20,276	1,439	0.97 (0.90–1.05), *p* = 0.452	1.02 (0.95–1.10), *p* = 0.591
Q3 (≥9.55– ≤ 17.56)	12.91	20,619	1,395	0.93 (0.87–1.00), *p* = 0.060	1.01 (0.93–1.09), *p* = 0.869
Q4 (≥17.67– ≤ 32.44)	23.79	20,928	1,449	0.96 (0.89–1.03), *p* = 0.289	1.03 (0.96–1.11), *p* = 0.425
Q5 (≥32.64)	50.24	19,506	1,399	0.98 (0.91–1.06), *p* = 0.615	1.04 (0.96–1.14), *p* = 0.301
				*p* for trend = 0.988	*p* for trend = 0.301
**Tomato catsup (g/day)**					
Q1 (≤ 0.11)	0	21,636	1,512	Reference group	Reference group
Q2 (≥0.13– ≤ 0.44)	0.17	19,575	1,310	1.00 (0.93–1.08), *p* = 0.898	1.02 (0.94–1.10), *p* = 0.656
Q3 (≥0.48– ≤ 1.15)	0.58	21,243	1,372	0.95 (0.88–1.02), *p* = 0.163	0.97 (0.90–1.05), *p* = 0.478
Q4 (≥1.20– ≤ 2.53)	1.99	19,486	1,494	0.96 (0.89–1.04), *p* = 0.308	0.98 (0.91–1.06), *p* = 0.567
Q5 (≥2.95)	5.06	19,897	1,473	1.02 (0.95–1.10), *p* = 0.568	1.00 (0.93–1.08), *p* = 0.935
				*p* for trend=0.324	*p* for trend=0.907
**Lycopene (mg/day)**					
Q1 (<2.79)	2.07	20,368	1,531	Reference group	Reference group
Q2 (≥2.79– <4.06)	3.42	20,367	1,367	0.89 (0.83–0.96), *p* = 0.002	0.91 (0.85–0.98), *p* = 0.016
Q3 (≥4.06– <5.61)	4.76	20,368	1,411	0.93 (0.86–1.00), *p* = 0.037	0.95 (0.88–1.03), *p* = 0.193
Q4 (≥5.61– <8.44)	6.74	20,367	1,346	0.88 (0.82–0.95), *p* = 0.001	0.89 (0.82–0.96), *p* = 0.004
Q5 (≥8.44)	12.06	20,367	1,506	0.98 (0.91–1.06), *p* = 0.617	0.95 (0.87–1.04), *p* = 0.261
				*p* for trend = 0.520	*p* for trend = 0.677

# *Adjusted for age (continuous) and sex (male vs. female)*.

* *Further adjusted for race (non-Hispanic White vs. Other), body mass index (BMI, continuous), education (≤ high school vs. ≥some college), smoking status (never vs. former ≤ 15 years since quit vs. former >15 years since quit vs. former year since quit unknown vs. current smoker ≤ 1 pack per day vs. current smoker >1 pack per day vs. current smoker intensity unknown), marital status (married vs. not married), randomization arm (screening group vs. control group), aspirin use (yes vs. no), history of hypertension (yes vs. no), history of diabetes (yes vs. no), history of stroke (yes vs. no), history of heart attack (yes vs. no), vegetables intake (continuous), fruit intake (continuous), alcohol drinking status (never vs. former vs. current), and total energy intake (continuous)*.

### Restricted Cubic Spline Model Analysis

**Figure 1:**
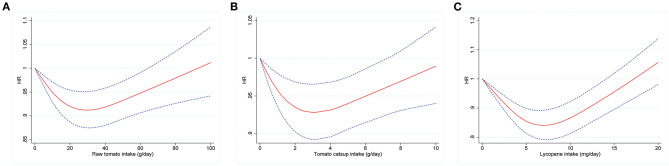
Dose-response analyses for the association between intakes of raw tomato **(A)**, tomato catsup **(B)**, or lycopene **(C)** and all-cause mortality were performed using restricted cubic spline model with 3 knots at 10th, 50th and 90th percentiles. Models were adjusted for age, sex, race, body mass index, education, smoking status, total energy intake, alcohol drinking status, marital status, randomization arm, aspirin use, history of hypertension, history of diabetes, history of stroke, history of heart attack, vegetables intake, and fruit intake. Red solid lines represent point estimates and blue dashed lines represent 95% confidence intervals (CIs).

There was a non-linear J-shaped association between raw tomato consumption and total mortality (magnitude of the relative reduction = 9%; nadir at 31 g/day; *P*-value for non-linear association <0.001; Figure 1A). A J-shaped association was also observed for tomato catsup (magnitude of the relative reduction = 7%; nadir at 3 g/d; *P* for non-linearity <0.001; Figure 1B) and lycopene (magnitude of the relative reduction = 16%; nadir at 7 mg/day; *P* for non-linearity <0.001; Figure 1C).

### Additional Analyses

The results of subgroup analyses are presented in Table 5. Tomato or lycopene intake remained consistently associated with reduced total mortality in almost all subgroups. An exception was represented by smoking status for tomato catsup. The association between tomato catsup intake and all-cause mortality risk was stronger in former cigarette smokers compared with that in current smokers (*P* for interaction = 0.035). In sensitivity analysis, results remained qualitatively similar after excluding events ascertained within 2 or 5 years (data not shown). Similar results were also obtained for total mortality when using ratio of tomato intake to total energy intake as exposure (raw tomato: Q5 vs. Q1; HR, 0.93 [95% CI, 0.89–0.97]; tomato catsup: Q5 vs. Q1; HR, 0.94 [95% CI, 0.90–0.97]; lycopene: Q4 vs. Q1; HR, 0.88 [95% CI, 0.84–0.92]).

**Table 5:** Subgroup analyses of the associations between tomato or lycopene intake and total mortality were performed based on sex, smoking status, and BMI.

**Variables**	**Group**	**Raw tomato**	***P* for interaction**	**Tomato catsup**	***P* for interaction**	**Lycopene**	***P* for interaction**
Male	Q1	Reference group	>0.05		>0.05		>0.05
	Q2	0.95 (0.91–1.00), *p* = 0.056		0.97 (0.91–1.03), *p* = 0.296		0.95 (0.90–1.01), *p* = 0.097	
	Q3	0.94 (0.89–0.99), *p* = 0.021		0.92 (0.87–0.98), *p* = 0.006		0.93 (0.88–0.99), *p* = 0.017	
	Q4	0.90 (0.86–0.96), *p* < 0.001		0.94 (0.90–0.99), *p* = 0.023		0.91 (0.86–0.97), *p* = 0.002	
	Q5	0.95 (0.90–1.00), *p* = 0.059		0.93 (0.88–0.98), *p* = 0.007		1.01 (0.95–1.08), *p* = 0.671	
Female	Q1	Reference group					
	Q2	0.97 (0.90–1.03), *p* = 0.308		0.92 (0.87–0.98), *p* = 0.004		0.88 (0.83–0.94), *p* < 0.001	
	Q3	0.91 (0.85–0.97), *p* = 0.004		0.89 (0.84–0.94), *p* < 0.001		0.87 (0.81–0.93), *p* < 0.001	
	Q4	0.92 (0.86–0.99), *p* = 0.017		0.93 (0.85–1.00), *p* = 0.064		0.85 (0.79–0.91), *p* < 0.001	
	Q5	0.96 (0.89–1.03), *p* = 0.235		0.94 (0.88–1.01), *p* = 0.110		0.95 (0.87–1.03), *p* = 0.192	
Never smokers	Q1	Reference group	>0.05		0.035		>0.05
	Q2	0.93 (0.87–0.99), *p* = 0.027		0.93 (0.87–0.99), *p* = 0.031		0.88 (0.83–0.94), *p* < 0.001	
	Q3	0.89 (0.83–0.95), *p* < 0.001		0.91 (0.85–0.97), *p* = 0.003		0.87 (0.81–0.93), *p* < 0.001	
	Q4	0.88 (0.82–0.94), *p* < 0.001		0.93 (0.87–1.00), *p* = 0.051		0.85 (0.79–0.92), *p* < 0.001	
	Q5	0.93 (0.87–1.01), *p* = 0.075		0.95 (0.89–1.02), *p* = 0.132		0.94 (0.87–1.02), *p* = 0.148	
Current smokers	Q1	Reference group					
	Q2	1.05 (0.96–1.16), *p* = 0.296		1.05 (0.94–1.16), *p* = 0.396		0.93 (0.84–1.03), *p* = 0.168	
	Q3	0.96 (0.86–1.07), *p* = 0.441		0.94 (0.84–1.04), *p* = 0.223		0.92 (0.82–1.03), *p* = 0.133	
	Q4	1.03 (0.92–1.14), *p* = 0.609		0.89 (0.80–0.99), *p* = 0.035		0.89 (0.79–1.00), *p* = 0.046	
	Q5	0.97 (0.87–1.09), *p* = 0.668		0.98 (0.88–1.09), *p* = 0.713		0.95 (0.84–1.08), *p* = 0.463	
Former smokers	Q1	Reference group					
	Q2	0.95 (0.89–1.01), *p* = 0.074		0.92 (0.87–0.98), *p* = 0.006		0.95 (0.89–1.01), *p* = 0.083	
	Q3	0.94 (0.89–1.00), *p* = 0.044		0.89 (0.84–0.95), *p* < 0.001		0.93 (0.87–0.98), *p* = 0.014	
	Q4	0.90 (0.84–0.95), *p* < 0.001		0.93 (0.88–0.98), *p* = 0.013		0.91 (0.85–0.97), *p* = 0.005	
	Q5	0.95 (0.89–1.01), *p* = 0.125		0.89 (0.84–0.95), *p* < 0.001		1.04 (0.97–1.12), *p* = 0.288	
BMI <25.0 kg/m^2^	Q1	Reference group	>0.05		>0.05		>0.05
	Q2	0.94 (0.88–1.01), *p* = 0.076		0.90 (0.84–0.96), *p* = 0.002		0.87 (0.82–0.93), *p* < 0.001	
	Q3	0.88 (0.82–0.94), *p* < 0.001		0.89 (0.84–0.96), *p* = 0.001		0.88 (0.82–0.94), *p* < 0.001	
	Q4	0.92 (0.85–0.98), *p* = 0.017		0.90 (0.84–0.97), *p* = 0.004		0.84 (0.77–0.90), *p* < 0.001	
	Q5	0.90 (0.83–0.97), *p* = 0.007		0.94 (0.87–1.01), *p* = 0.109		0.96 (0.88–1.05), *p* = 0.343	
BMI ≥25.0 kg/m^2^	Q1	Reference group					
	Q2	0.97 (0.92–1.02), *p* = 0.220		0.97 (0.92–1.02), *p* = 0.265		0.95 (0.91–1.00), *p* = 0.069	
	Q3	0.95 (0.91–1.00), *p* = 0.053		0.92 (0.88–0.97), *p* = 0.002		0.93 (0.88–0.98), *p* = 0.008	
	Q4	0.91 (0.87–0.96), *p* < 0.001		0.96 (0.91–1.01), *p* = 0.087		0.92 (0.87–0.97), *p* = 0.004	
	Q5	0.98 (0.93–1.04), *p* = 0.490		0.95 (0.90–1.00), *p* = 0.037		1.03 (0.97–1.09), *p* = 0.336	

*BMI, body mass index*.

## Discussion

In this large, adult, US population, moderate intakes of raw tomato, tomato catsup, and lycopene were associated with reduced risks of all-cause and CVD mortality. Moderate lycopene intake was also inversely associated with cancer mortality. The associations of tomato and lycopene intake with all-cause mortality followed a non-linear J-shaped curve.

The observed protective associations of moderate tomato and lycopene consumption with total and CVD deaths were in line with a previous prospective study using data from the US National Health and Nutrition Examination Survey (NHANES) showing evidence of an inverse relationship between tomato or lycopene consumption and all-cause and CVD mortality (14). Our findings of null association between tomato intake and cancer mortality were not in accordance with Mazidi's study (20), which found that both tomato and lycopene intake were inversely related to cancer mortality based on NHANES data. By contrast, we only observed that moderate lycopene intake was significantly associated with cancer mortality.

In our study, we examined the impact of specific types of tomatoes (i.e., raw tomato and tomato catsup) on mortality. This was in contrast to a previous prospective study on this topic, which only examined associations with total tomato intake (14). Different types of tomatoes may have differential effects on health outcomes. Fraser et al. (21) reported that there was no relationship between intake of raw tomatoes and prostate cancer risk. However, a statistically significant multivariate-adjusted relationship between the intake of canned and cooked tomatoes and prostate cancer risk was observed. One bladder cancer study also found an inverse relationship with cooked, but not raw, tomatoes (22). Potential mechanisms by which cooking affects the association between tomato intake and cancer risk include changes in availability of some nutrients, destruction of digestive enzymes, and alteration of the structure and digestibility of food (23). In our study, both raw tomatoes and tomato catsup were statistically significantly inversely associated with total and CVD mortality.

Emerging evidence has suggested that tomato or lycopene intake may exert beneficial effects on human health, including some cancers (prostate, liver and stomach) (12, 13, 24), metabolic syndrome (8), and CVD incidence (5). To the best of our knowledge, this is the first cohort study that has reported a J-shaped relationship between tomato or lycopene intake and health outcome. The possible underling mechanism is not clear and warrants further investigation. The findings of our study suggested that moderate consumption of tomato or lycopene was enough to reduce the all-cause and CVD deaths.

When we stratified results by smoking status, the association between tomato catsup intake and all-cause mortality risk was stronger in former smokers compared with that in current smokers (*P* for interaction = 0.035), implying the potential residual confounding of smoking behavior. Thus, in multivariate analysis, we categorified the smoking status in more details.

Several potential mechanisms could explain the beneficial effects of moderate tomato or lycopene consumption on health, including strong antioxidant capacity to protect against oxidative stress (25), cholesterol reduction, modulation of inflammatory markers, metabolism to retinoids, and antiangiogenic effects (26). Interestingly, treatment with lycopene could reduce the formation of advanced glycation end products in HK-2 cells and in rat kidneys (27). Enhanced generation and accumulation of advanced glycation end products have been associated with an increased risk for CVD complications (28). Finally, various intervention trials have found beneficial effects on CVD risk markers, although the conflicts still exist (29).

The major strengths of this study included a large sample size of participants; a prospective cohort design; detailed information on diet and potential risk factors for deaths; and analyses on both tomato and lycopene intake. However, several limitations should be acknowledged. First, given the observational nature of our investigation, causality can only be suggested and residual confounding cannot be fully ruled out. Second, the vast majority of participants analyzed in this study were non-Hispanic Whites, which may limit its generalizability to other populations. Third, the J-shaped association may indicate the potential residual confounding of variables not included in the model (e.g., social-economic status). It is also possible that moderate tomato intake represents certain types of dietary pattern or intake of a good variety of vegetables. Lastly, participants' information was collected at baseline only and the exposures could have changed during the follow-up period.

In conclusion, in this large nationally representative sample of US adult population, intakes of raw tomato, tomato catsup and lycopene were associated with lower risks of all-cause and CVD mortality. Moderate consumption of lycopene was also related with a reduced cancer mortality. Further clinical studies and dietary intervention studies are warranted to confirm our premilitary findings.

## Data Availability Statement

The datasets presented in this study can be found in online repositories. The names of the repository/repositories and accession number(s) can be found at: https://cdas.cancer.gov/datasets/plco/.

## Ethics Statement

The studies involving human participants were reviewed and approved by National Cancer Institute. The patients/participants provided their written informed consent to participate in this study.

## Author Contributions

XX contributed to the conception or design of the work. XX, YZ, and SL contributed to the acquisition, analysis, or interpretation of data for the work. XX and SL drafted the manuscript. YZ critically revised the manuscript. All authors gave final approval and agree to be accountable for all aspects of work ensuring integrity and accuracy.

## Conflict of Interest

The authors declare that the research was conducted in the absence of any commercial or financial relationships that could be construed as a potential conflict of interest.
