# Supplementary material for: Dietary Intake of Tomato and Lycopene and Risk of All-Cause and Cause-Specific Mortality: Results From a Prospective Study
Source: Front Nutr. 2021 Jul 5;8:684859. doi: 10.3389/fnut.2021.684859 (PMC8287057; doi:10.3389/fnut.2021.684859)
Supplement: Supplementary file 1 [file Table_1.DOCX]

**Supplemental Table S1 Main characteristic of participants included in this study by tomato catsup intake**

| **Variables** | **Q1 (n=21636)** | **Q2 (n=19575)** | **Q3 (n=21243)** | **Q4 (n=19486)** | **Q5 (n=19897)** | **p-value** |
| --- | --- | --- | --- | --- | --- | --- |
| Age (y), mean (SD) | 63.0 (5.4) | 62.7 (5.3) | 62.4 (5.3) | 62.2 (5.2) | 61.7 (5.1) | <0.001 |
| Sex (n, %) |  |  |  |  |  |  |
| Male | 8038 (37.2%) | 5884 (30.1%) | 7849 (37.0%) | 14698 (75.4%) | 13064 (65.7%) | <0.001 |
| Female | 13598 (62.8%) | 13690 (69.9%) | 13393 (63.0%) | 4787 (24.6%) | 6831 (34.3%) |  |
| Smoking status (n, %) |  |  |  |  |  |  |
| Never | 10585 (48.9%) | 9825 (50.2%) | 10741 (50.6%) | 8403 (43.1%) | 9042 (45.5%) | <0.001 |
| Current | 1974 (9.1%) | 1729 (8.8%) | 1844 (8.7%) | 1834 (9.4%) | 2031 (10.2%) |  |
| Former | 9074 (41.9%) | 8017 (41.0%) | 8652 (40.7%) | 9243 (47.4%) | 8818 (44.3%) |  |
| Education (n, %) |  |  |  |  |  |  |
| ≤High school | 8914 (41.2%) | 8194 (41.9%) | 8938 (42.1%) | 7860 (40.3%) | 9061 (45.5%) | <0.001 |
| ≥Some college | 12682 (58.6%) | 11346 (58.0%) | 12260 (57.7%) | 11584 (59.4%) | 10789 (54.2%) |  |
| BMI (n, %) |  |  |  |  |  |  |
| <25.0 kg/m^2^ | 8587 (39.7%) | 7739 (39.5%) | 7641 (36.0%) | 5489 (28.2%) | 5023 (25.2%) | <0.001 |
| ≥25.0 kg/m^2^ | 12731 (58.8%) | 11597 (59.2%) | 13328 (62.7%) | 13735 (70.5%) | 14613 (73.4%) |  |
| Race (n, %) |  |  |  |  |  |  |
| White, Non-Hispanic | 19365 (89.5%) | 17514 (89.5%) | 19333 (91.0%) | 17921 (92.0%) | 18464 (92.8%) | <0.001 |
| Other | 2263 (10.5%) | 2053 (10.5%) | 1900 (8.9%) | 1558 (8.0%) | 1424 (7.2%) |  |
| Alcohol drinking status (n, %) |  |  |  |  |  |  |
| Never | 2353 (10.9%) | 2230 (11.4%) | 2230 (10.5%) | 1484 (7.6%) | 1827 (9.2%) | <0.001 |
| Former | 3470 (16.0%) | 2858 (14.6%) | 2935 (13.8%) | 2617 (13.4%) | 2889 (14.5%) |  |
| Current | 15092 (69.8%) | 13924 (71.1%) | 15471 (72.8%) | 14909 (76.5%) | 14664 (73.7%) |  |
| Total energy intake (kcal/d), mean (SD) | 1540.0 (660.5) | 1521.3 (622.6) | 1629.6 (637.0) | 1897.6 (730.9) | 2128.7 (827.5) | <0.001 |

Y, year; SD, standard deviation; BMI, body mass index
