# Supplementary material for: Dietary Intake of Tomato and Lycopene and Risk of All-Cause and Cause-Specific Mortality: Results From a Prospective Study
Source: Front Nutr. 2021 Jul 5;8:684859. doi: 10.3389/fnut.2021.684859 (PMC8287057; doi:10.3389/fnut.2021.684859)
Supplement: Supplementary file 2 [file Table_2.DOCX]

**Supplemental Table S2 Main characteristic of participants included in this study by lycopene intake**

| **Variables** | **Q1 (n=20368)** | **Q2 (n=20367)** | **Q3 (n=20368)** | **Q4 (n=20367)** | **Q5 (n=20367)** | **p-value** |
| --- | --- | --- | --- | --- | --- | --- |
| Age (years), mean (SD) | 63.4 (5.4) | 62.7 (5.3) | 62.3 (5.2) | 61.9 (5.2) | 61.8 (5.1) | <0.001 |
| Sex (n, %) |  |  |  |  |  |  |
| Male | 8027 (39.4%) | 8720 (42.8%) | 9547 (46.9%) | 10701 (52.5%) | 12538 (61.6%) | <0.001 |
| Female | 12340 (60.6%) | 11647 (57.2%) | 10819 (53.1%) | 9666 (47.5%) | 7827 (38.4%) |  |
| Smoking status (n, %) |  |  |  |  |  |  |
| Never | 10085 (49.5%) | 9992 (49.1%) | 9803 (48.1%) | 9639 (47.3%) | 9077 (44.6%) | <0.001 |
| Current | 2046 (10.0%) | 1822 (8.9%) | 1717 (8.4%) | 1789 (8.8%) | 2038 (10.0%) |  |
| Former | 8232 (40.4%) | 8550 (42.0%) | 8842 (43.4%) | 8932 (43.9%) | 9248 (45.4%) |  |
| Education (n, %) |  |  |  |  |  |  |
| ≤High school | 9557 (46.9%) | 8575 (42.1%) | 8372 (41.1%) | 8213 (40.3%) | 8250 (40.5%) | <0.001 |
| ≥Some college | 10765 (52.9%) | 11753 (57.7%) | 11953 (58.7%) | 12121 (59.5%) | 12069 (59.3%) |  |
| BMI (n, %) |  |  |  |  |  |  |
| <25.0 kg/m^2^ | 8031 (39.4%) | 7535 (37.0%) | 7018 (34.5%) | 6260 (30.7%) | 5635 (27.7%) | <0.001 |
| ≥25.0 kg/m^2^ | 12056 (59.2%) | 12555 (61.6%) | 13096 (64.3%) | 13881 (68.2%) | 14416 (70.8%) |  |
| Race (n, %) |  |  |  |  |  |  |
| White, Non-Hispanic | 17305 (85.0%) | 18654 (91.6%) | 18955 (93.1%) | 19002 (93.3%) | 18681 (91.7%) | <0.001 |
| Other | 3055 (15.0%) | 1706 (8.4%) | 1407 (6.9%) | 1360 (6.7%) | 1670 (8.2%) |  |
| Alcohol drinking status (n, %) |  |  |  |  |  |  |
| Never | 2571 (12.6%) | 2090 (10.3%) | 1951 (9.6%) | 1770 (8.7%) | 1742 (8.6%) | <0.001 |
| Former | 3571 (17.5%) | 2921 (14.3%) | 2701 (13.3%) | 2710 (13.3%) | 2866 (14.1%) |  |
| Current | 13467 (66.1%) | 14804 (72.7%) | 15217 (74.7%) | 15373 (75.5%) | 15199 (74.6%) |  |
| Total energy intake (kcal/d), mean (SD) | 1266.9 (516.6) | 1490.2 (538.1) | 1693.6 (579.8) | 1951.7 (664.6) | 2290.3 (861.1) | <0.001 |

Y, year; SD, standard deviation; BMI, body mass index
